# Renal injury and hepatic effects from the methylimidazolium ionic liquid M8OI in mouse

**DOI:** 10.1016/j.ecoenv.2020.110902

**Published:** 2020-10-01

**Authors:** Alistair C. Leitch, Tarek M. Abdelghany, Alex Charlton, Justina Grigalyte, Fiona Oakley, Lee A. Borthwick, Lee Reed, Amber Knox, William J. Reilly, Loranne Agius, Peter G. Blain, Matthew C. Wright

**Affiliations:** a Health Protection Research Unit, Wolfson Building, Newcastle University, Newcastle Upon Tyne, NE2 4AA, United Kingdom; b Institute Translational and Clinical Research, Level 4 Leech, Newcastle University, Newcastle Upon Tyne, NE2 4HH, United Kingdom; c Department of Pharmacology and Toxicology, Faculty of Pharmacy, Cairo University, Kasr El-Aini St., Cairo, 11562, Egypt; d School of Natural and Environmental Sciences, Bedson Building, Newcastle University, NE1 8QB, United Kingdom; e Newcastle Fibrosis Research Group, Biosciences Institute, Newcastle University, Newcastle Upon Tyne, NE2 4HH, United Kingdom

**Keywords:** C8mim, Kidney, kim1, Liver, Fibrosis, Glycogen, ALT, alanine aminotransferase, ALP, alkaline phosphatase, ANIT, α-naphthylisothiocyanate, CK-19, cytokeratin 19, CLPZ, chlorpromazine, COOH7IM, 1-(7-carboxyheptyl)-3-methyl-1H-imidazol-3-ium, ER, estrogen receptor, H & E, haematoxylin and eosin, HO8IM, 1-(8-Hydroxyoctyl)-3-methyl-imidazolium, i.p., intraperitoneal, kim1, kidney injury molecule 1, M8OI, 1-octyl-3-methylimidazolium, OA, okadaic acid, PAS, periodic acid stained, PBC, primary biliary cholangitis, PBS, phosphate buffered saline

## Abstract

The ionic liquid 1-octyl-3-methylimidazolium (M8OI) has been found in the environment and identified as a hazard for triggering the liver disease primary biliary cholangitis (PBC). Given limited toxicity data for M8OI and other structurally-related ionic liquids, target organs for M8OI toxicity were examined. Adult male C57Bl6 mice were acutely exposed to 0–10 mg/kg body weight M8OI via 2 intraperitoneal injections (time zero and 18 h) and effects examined at 24 h. At termination, tissue histopathology, serum and urinary endpoints were examined. No overt pathological changes were observed in the heart and brain. In contrast, focal and mild to multifocal and moderate degeneration with a general trend for an increase in severity with increased dose was observed in the kidney. These changes were accompanied by a dose-dependent increased expression of Kim1 in kidney tissue, marked elevations in urinary Kim1 protein and a dose-dependent increase in serum creatinine. Hepatic changes were limited to a significant dose-dependent loss of hepatic glycogen and a mild but significant increase in portal tract inflammatory recruitment and/or fibroblastic proliferation accompanied by a focal fibrotic change. Cultured mouse tissue slices reflected these *in vivo* effects in that dose-dependent injury was observed in kidney slices but not in the liver. Kidney slices accumulated higher levels of M8OI than liver slices (e.g. at 10 μM, greater than 4 fold) and liver slices where markedly more active in the metabolism of M8OI. These data indicate that the kidney is a target organ for the toxic effects of M8OI accompanied by mild cholangiopathic changes in the liver after intraperitoneal administration.

## Highlights

• The ionic liquid M8OI [C8mim] has been found in the environment.
• Mice were exposed acutely over 24 h to M8OI via i.p. injection.
• The kidney was the target organ for toxicity, heart and brain appeared normal.
• Hepatic changes were limited to glycogen depletion and mild portal tract changes.
• Kidney tissue slices accumulated higher levels of M8OI than liver tissue slices.

## 1 Introduction

**Fig. 1:**
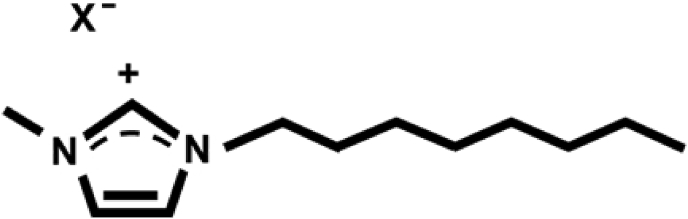
**Chemical structure of M8OI.** The M8OI anion charge is balanced by a cation (X^−^) and is readily available as the Cl^−^ salt (CAS # 64697-40-1) as described in these studies. Other salts, e.g. bromide (CAS # 61545-99-1); a hexafluorophosphate (CAS # 304680-36-2); a tetrafluoroborate (CAS # 244193-52-0) and a trifluoromethanesulfonate (CAS # 403842-84-2) are also available commercially.

A recent investigation into chemicals in soils around a land-fill waste site identified - in selected sampling sites - the presence of a chemical initially identified based on toxicity to a liver progenitor cell line (Probert et al., 2018). The chemical structure was identified as the ionic liquid 1-octyl-3-methylimidazolium (M8OI, also referred to as C8mim, for structure see Fig. 1) and authentic pure M8OI recapitulated the toxic effects of M8OI in the hepatic progenitor (and human cholangiocytes): an inhibition of mitochondrial oxidative phosphorylation and apoptosis (Probert et al., 2018). Subsequent re-analysis of soil samples indicated that the M8OI levels in the most contaminated sites where at present in the aqueous soil extracts and originating soils at 13 mM and 0.3 mg/kg respectively (Leitch et al., 2020).

The term “ionic liquids” encompasses a diverse range of chemicals characterised as having low volatilities at ambient temperatures (Welton, 2018). The low volatility of ionic liquids is widely considered likely to benefit the environment through reduced evaporation during use in a variety of industrial applications and accordingly, they are often proposed to be a replacement for many currently used solvents (Welton, 2018).

A number of characteristics of ionic liquids could be of concern (Oskarsson and Wright, 2019). Ionic liquid chemicals such as M8OI contain structural moieties not present in biological molecules (i.e. the imidazolium group), which may render them relatively resistant to degradation in the environment. In this respect, the bromide salt of M8OI has been shown to resist biodegradation and to be only partially mineralized (Docherty et al., 2007). Ionic liquids such as M8OI are also lipophilic ionic molecules, which may render them both readily absorbed and biologically (toxicologically) active in biological systems. In our laboratory, M8OI was shown to act as an inhibitor of mitochondrial oxidative phosphorylation in both the B-13 liver progenitor cell line and human cholangiocytes (Probert et al., 2018).

The potential adverse environmental effects of M8OI have been investigated using a variety of model systems. M8OI (as the Br salt) has been shown to be acutely toxic to frogs (*R. nigromaculata*) during early embryonic development (Li et al., 2009); (as the hexafluorophosphate salt) to irreversibly inhibit the germination of wheat (Liu et al., 2014); (as the Cl-salt) affect membrane permeability, cell morphology and growth of green algae (Liu et al., 2015); (as the Br-salt) inhibit photosynthesis and cell growth in the marine diatom (phytoplankton) S. costatum (Deng et al., 2016); (as the Br-salt) to be genotoxic to planarians (flatworms, using D. japonica) (Zhang et al., 2016) and toxic to fish (*P. dabryanus*), with the liver as the target (Nan et al., 2016).

Few limited studies have been completed in mammalian species. M8IO (as the Br-salt) has been shown to be cytotoxic to the human hepatoma HepG2 cell line (Li et al., 2015; Ma and Li, 2018). An examination of a variety of toxic endpoints suggested that an induction of reactive oxygen species was an early effect of exposure and that the mode of cell death was apoptotic based on induction of caspase activities (Li et al., 2015; Ma et al., 2018). The acute toxic effects of M8OI (as the Br-salt) has also been examined in mice. The study was limited to potential adverse effects up to 24 h after i. p. administration and the authors calculated an LD_50%_ of 35.7 mg/kg body weight (Yu et al., 2008). Ten hours after administration, the authors report histopathological changes in the liver. More recently, oral exposure to M8OI and a related ionic liquid (both as the Cl-salt) in mice have been shown to modulate the gut microbiota (Young et al., 2020).

We have recently proposed that ionic liquids with a structural relationship to M8OI could adversely affect the liver and trigger cholestatic-related disease such as primary biliary cholangitis (PBC) through several pathways acting in concert (Probert et al., 2018). These include a sensitivity of an hepatic progenitor population to the direct mitochondrial toxicity of M8OI; its hepatic metabolism to a carboxylic acid that can replace lipoic acid in 2 oxo acid dehydrogenase enzymes such as the E2 component of the pyruvate dehydrogenase complex; a cholestatic injury and an oestrogenic effect that exacerbates cholestatic injury and/or inflammatory fibrotic progression (Meyer et al., 2017; Leitch et al., 2018, Wallace et al., 2008).

In order to begin to test this hypothesis, the identity of the likely target for acute toxic effects was assessed through near-systemic (i.p. administration) exposure. To avoid any potential interactions of counter-ion toxicity, the effects of the chloride salt (M8OI.Cl, i.e. the common physiological anion) was used. Although oral exposure is the most likely exposure route for M8OI in man, given the limited data on mammalian toxicity of M8OI overall and in particular, *in vivo*, identification of target organs for toxicity is most effectively achieved through a short term study with assured systemic exposure. We show that the kidney is a target organ for the toxic effects of M8OI and that the liver is also affected based on glycogen depletion and mild but significant increases in portal tract inflammatory/fibroblastic cell recruitment accompanied by a focal fibrotic change. *In vitro* precision cut tissue slice experiments recapitulate the *in vivo* toxic effects of M8OI and suggest that high accumulation of M8OI by the kidney is a significant factor in this organ being a target for toxic effects.

## 2 Material and methods

### 2.1 Materials

The M8OI (as the Cl^−^ salt; purity >97%) and alpha-naphthylisothiocyanate (ANIT; purity 95%) were purchased Sigma (Poole, UK). Rabbit anti-kidney injury molecule 1 (Kim1) (cat # Ab47635); anti-cytokeratin 19 (CK-19) (Cat # Ab84632); rabbit anti-vimentin (Cat # Ab92547) and anti-α-smooth muscle actin (Cat # Ab32575) antibodies were purchased from Abcam. Rabbit anti-active caspase 3 antibodies (Cat #G7481) were purchased from Promega.

### 2.2 Animal study

**Fig. 2:**
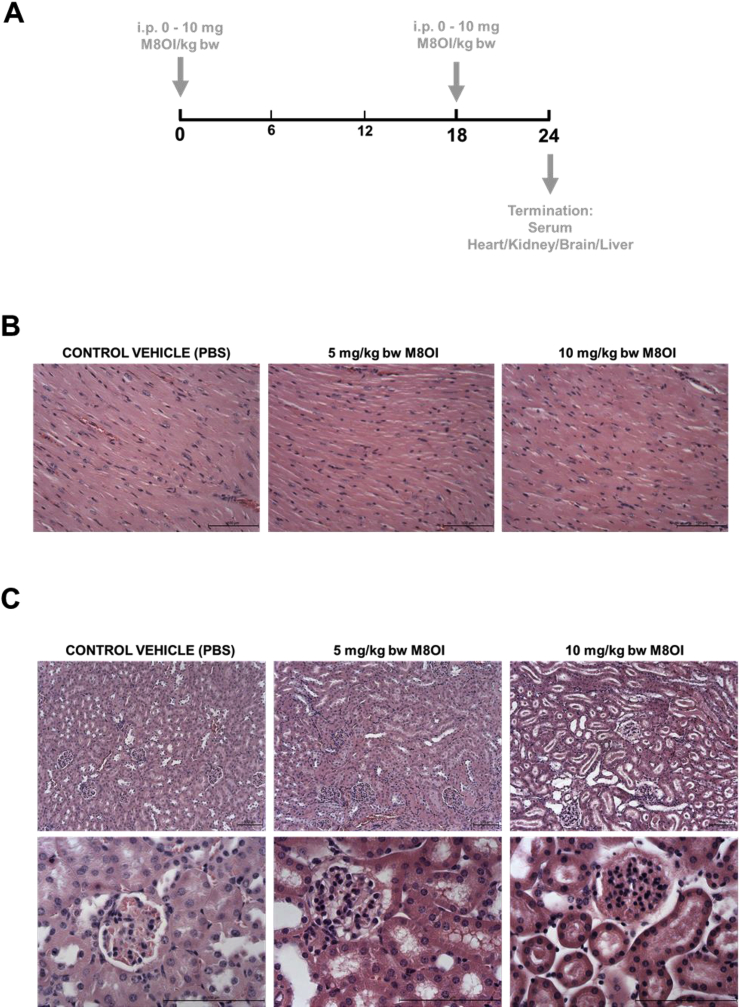

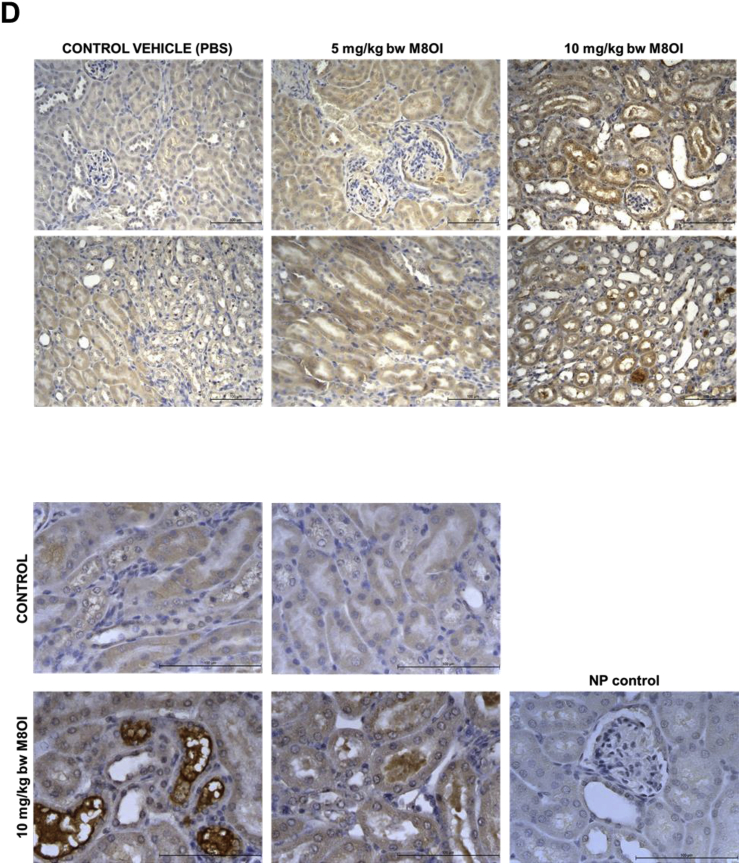
**Cardiac and renal effects of M8OI exposure *in vivo*. A**, schematic diagram of the time course for administration of M8OI and termination. **B**, typical histopathological views of mouse heart, haematoxylin and eosin stained sections. **C**, typical histopathological views of mouse kidney, haematoxylin and eosin stained kidney sections. Upper panels at low magnification, lower panels higher magnification showing staining and morphological changes in both the glomerular and duct regions. **D**, typical Kim1 immunohistochemistry in kidney sections showing an increase in stain intensity with increasing dose of M8OI. Lower panels show higher magnification views and no primary antibody control (NP control), a section treated identically to others sections but without the addition of the anti-Kim1 antibody during primary antibody incubation step.

Male C57Bl6 mice were purchased from Charles River and housed in the Comparative Biology Centre at Newcastle University. Only male mice were used in this study in order to reduce the number of animals used and to avoid the potential complications of variations in female sex hormone levels. Mice (32 weeks of age; 30 ± 5 g body weight) were randomly assigned to one of 3 dose groups, control vehicle, 5 mg/kg body weight and 10 mg/kg body weight. As a qualitative study for the determination of any potential adverse effects of M8OI, 3–5 animals were assigned to each group in order to reduce the number of animals used whilst retaining the option to apply statistical tests on endpoints. Mice were dosed by i. p. injection with M8OI in sterile phosphate buffered saline (137 mM NaCl, 27 mM KCl, 100 mM phosphate pH 7.4, PBS) vehicle at the beginning of the study and at 18 h prior to cervical dislocation at 24 h and removal of tissues, blood and urine (by direct extraction from the bladder) for analyses (see Fig. 2a). Mice in the control group were administered sterile PBS vehicle only. It should be noted that the only available study with any ionic liquid in a mammalian species is with the bromide salt of M8OI (M8OI [Br-]), using a single i. p. injection in mice at a dose up to 40 mg/kg body weight (Yu et al., 2008). The authors report an LD_50%_ of 35.7 mg/kg for M8OI [Br^−^] suggesting that any hepatic effects may have been significantly influenced by non-specific effects of multiple organ failure. This short-term systemic exposure protocol (licenced by the UK Home Office with Local Ethics Committee approval) was adopted in order to maximise detection of target organ effects whilst reducing the number of animals used and reducing potential for severe adverse effects.

Archived mouse tissue from female adult mice treated with α-naphthylisothiocyanate (ANIT) was used as positive controls for the presence of active caspase 3 in the liver, in order to reduce the number of animals used in this study. Mice were orally gavaged with 30 mg/kg body weight with ANIT in olive oil (4 mL/kg body weight), control vehicle mice received olive oil only. Mice were subjected to cervical dislocation 16 days later prior to removal of livers (once evidence of hepatocellular necrosis had subsided but hepatic inflammation remained elevated – data not included).

### 2.3 Liver cell isolations

Mouse hepatocytes were prepared from untreated male C57Bl6 mice by a two-step collagenase procedure as previously described (Leitch et al., 2018). In brief, mice were terminally anaesthetised using isoflurane, administered heparin followed by laparotomy. The liver was perfused via cannulation into the heart at 5 ml/min for 5 min with calcium-free Hank's buffer followed by 20 min with Hank's buffer containing 0.1 mg/ml collagenase (Sigma, C5138). The liver was then removed and cells dispersed by agitation into Hank's buffer. After filtration to remove clumps, cells were sedimented at 50×*g* for 3 min. Pelleted cells were re-suspended in Hank's buffer and sedimented again at 50×*g* for 3 min prior to final suspension in William's Medium E supplemented with 10 nM dexamethasone, 1 μg/ml insulin, 100 units/ml penicillin, 100 μg/ml streptomycin and 10% FCS. Cells were seeded onto collagen-coated plastic cultureware (Greiner) and cultured in an humidified incubator at 37 °C and 5% CO_2_ in air overnight, prior to replenishment with medium without FCS.

Assessments of cell viability (trypan blue exclusion) and endpoints for apoptosis (active caspase 3/7 activity; active caspase 3 immunocytochemistry; DNA nucleosomal ladders) have been described previously (Wright et al., 2001; Orr et al., 2004).

### 2.4 Tissue slicing and culture

Tissue liver and kidney 8-mm cores (using a Stiefel biopsy punch, Medisave, Weymouth, UK) were prepared, submerged in 3% low gelling temperature agarose and sliced using a Leica VT1200S vibrating blade microtome (Leica Biosystems, Milton Keynes, UK) essentially as outlined (Paish et al., 2019). In brief, liver slices were cultured in a modified tissue culture plate (BioR plate) and rocked on the bioreactor platform (patent PCT/GB 2016/053310) in Williams medium E supplemented with 1% penicillin/streptomycin and l-glutamine, 1 × insulin transferrin-selenium X, 2% fetal bovine serum (Thermo Fisher Scientific, Cramlington, UK) and 100 nM dexamethasone at 37 °C, in a humidified atmosphere in air supplemented with 5% CO_2_. Kidney slices were generated as described for liver slices and cultured in DMEM-F12 (Gibco) supplemented with REGM SingleQuot Kit (Lonza), 1% penicillin/streptomycin and L-glutamine.

After an overnight recovery period, media were replenished with additions indicated, and slices cultured for up to a further 6 h (for metabolic studies) or 24 h (to examine any potential toxic effects).

### 2.5 M8OI and M8OI metabolite determinations in tissues

M8OI and its metabolites were quantified by standard multiple reaction monitoring (MRM) techniques using a Waters Xevo TQ-S Triple Quadrupole Mass-Spectrometer coupled to a Waters Acquity UPLC. Chromatographic separation was achieved by gradient elution with a ACE Excel 3 C18-PFP (150 mm × 2.1 mm; 3 μm particle size) chromatography column equipped with an ACE Excel UPLC Pre-Column filter guard column, with (A) 0.1% formic acid in water and (B) 0.1% formic acid in acetonitrile as mobile phase, at a flow rate of 250 μL/min 10% B at t = 0 min, then to 50% B at t = 5 min, and 90% B at t = 8 min. Then returning to 10% B at t = 9 min, and holding for 1 min. The column temperature was 40 °C, with 1 μL injection volume per sample. MRM transitions for M8OI, HO8IM and COOH7IM were 195.2 -> 83.1, 211.2 -> 83.1 and 225.2 -> 83.1 respectively, with a collision energy of 20eV.

### 2.6 Clinical chemistry

Tail vein blood was collected and serum prepared as outlined below for clinical chemistry to monitor on-going liver injury in ANIT-treated mice. At termination, blood was collected and allowed to clot in a 1.5 ml eppendorf prior to centrifugation at 13000 rpm for 1 min and collection of the serum supernatant. Serum alanine aminotransferase (ALT) and alkaline phosphatase (ALP) activities and serum glucose and creatinine concentrations were determined as previously outlined (Meyer et al., 2017).

A qualitative examination for urinary haemoglobin, pH and protein were performed using Hema-combistix reagent strips for urinalysis (Siemens). Urinary total protein levels were determined using the Biorad protein assay using BSA as standard. Urinary Kim1 levels were estimated by Western blotting. Urine samples were mixed 1:1 with SDS-containing thiol reducing loading buffer (62.5 mM Tris pH 6.8, 2% w/v SDS, 10% v/v glycerol, 0.004% w/v bromophenol blue and 10 mM dithiothreitol) prior to Western blotting essentially as described (Fairhall et al., 2018).

### 2.7 Tissue pathology and immunohistochemistry

Tissues (brain, heart, liver, kidney, pancreas) were fixed in 10% formalin in PBS, processed, embedded in wax and 4 μm sections stained with haematoxylin and eosin or sirius red (followed by haematoxylin) essentially as previously outlined (Meyer et al., 2017). Liver sections were also periodic acid stained (PAS) with or without prior treatment with diastase essentially as described (Fairhall et al., 2018), for the detection of glycogen. Sections were also subjected to immunohistochemical analyses for vimentin, cytokeratin 19 (CK-19) and α-smooth muscle actin as previously outlined (Marek et al., 2005). For Kim1, kidney sections were microwaved for 20 min in 0.1 M sodium citrate to retrieve antigens prior to cooling and incubation with 1/200 anti-Kim antibody overnight, followed by incubation with anti-rabbit IgG (HRP conjugated) and subsequent visualisation using DAB.

PAS-stained sections were scanned on a Leica SCN400 slidescanner and stain intensity and area were determined after manually setting the baseline to extract a staining concentration per measurement unit value. Data are presented as mean staining intensity per pixel. Randomly selected portal tract inflammatory/fibroblastic cell recruitment was determined from haematoxylin and eosin (H & E) stained liver sections by an examiner blinded to the treatment group.

### 2.8 Tissue biochemistry

Acid-soluble and hydrolysable reducing sugar tissue concentrations were determined as previously described, using rabbit liver glycogen as standard (Fairhall et al., 2018).

## 3 Results

### 3.1 The kidney is a target organ for the toxic effects of M8OI in mice after i.p. administration

**Fig. 3:**
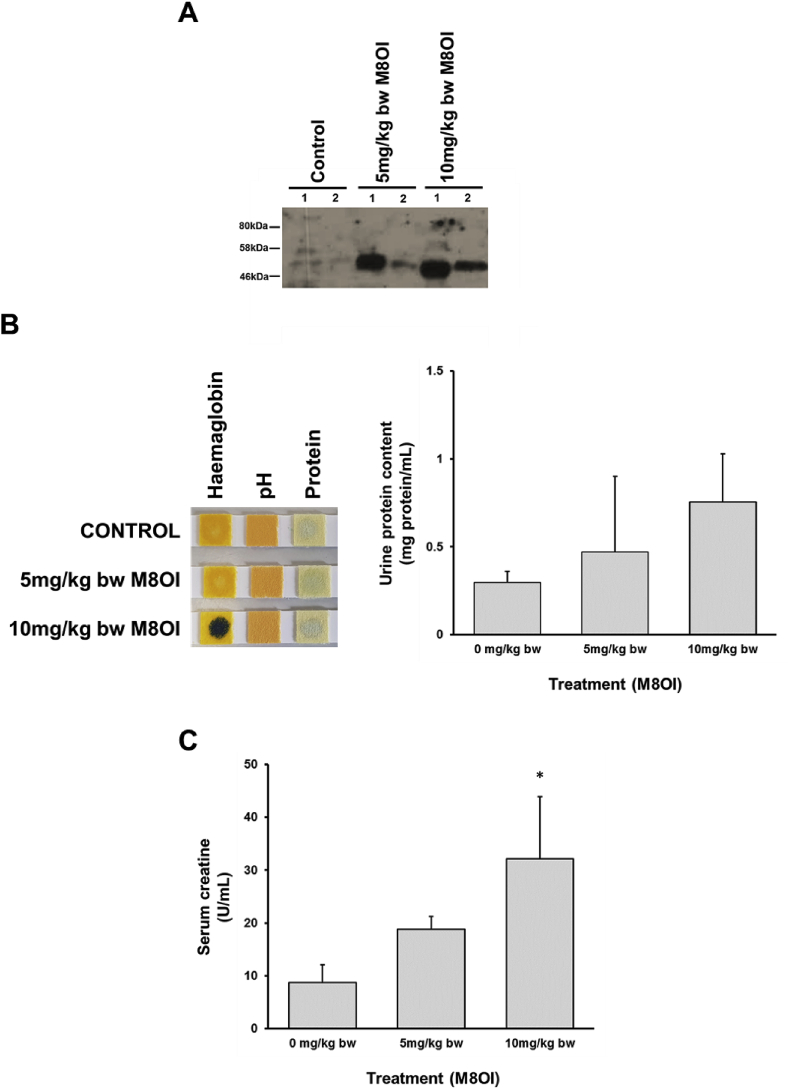
**Urinary and serum markers of renal injury are raised in M8OI-treated mice. A**, Western blot for Kim1 protein in urine from the mice treated with the indicated dose of M8OI, results typical of at least 3 mice/group. Each lane contains 7.5 μl urine/lane. **B**, left panel, typical urinary dipstick response to the indicated M8OI dose; right panel, urinary protein content. Data are the mean and SD of at least 3 animal urine samples (i.e. not technical replicates; sufficient urine was not obtained from all animals in the study). **C**, serum creatinine concentration. Data are the mean and SD of at least 3 separate animal serum samples (i.e. not technical replicates) per group. *Significantly different (P > 0.95) from control using one way ANOVA followed by bonferroni post hoc test.

In order to establish whether there is target organ(s) injury associated with systemic exposure, M8OI was administered by i. p. injection twice over a 24 h period (for scheme, see Fig. 2a). Since in vitro studies in a cell line indicate that M8OI targets mitochondrial oxidative phosphorylation (Probert et al., 2018), tissues associated with an high energy requirement (heart, kidney, brain) in addition to liver were examined. No overt pathological changes were observed in the heart (Fig. 2b) as well as the brain and pancreas (data not shown). In contrast, histopathological changes were seen in the kidneys of mice treated with M8OI (Fig. 2c). In H & E stained sections, there was clear evidence of focal and mild degeneration (in 2 out of 4 animals) and multifocal and moderate degeneration (in 1 animal) in the high dose group (10 mg/kg body weight) predominantly localised to the cortex. The necrotic changes were evident in glomeruli and in both proximal and distal tubules (characterised by hydropic degeneration and desquamation of cells from the tubules). Although these changes were variable between individuals within both the 5 mg/kg body weight and 10 mg/kg body weight groups and changes were patchy, there was a general trend for an increase in severity with an increased dose of M8OI. Fig. 2d demonstrates that there was a dose-dependent increased expression of Kim1 in kidney tissue, most notably in the ducts, with evidence for its presence within the ducts, suggestive of injury and shedding of Kim1 protein into the urine, a common feature with renal injury (Han et al., 2002). Examination of urinary protein Kim1 levels (Fig. 3a) indicates marked elevations in Kim1 protein. Fig. 3b demonstrates detectable levels of haemoglobin and a dose-dependent trend for an increase in urinary protein concentration with M8OI dose, though lacking statistical significance. These changes in combination with a dose-dependent increase in serum creatinine (Fig. 3c) – an indirect biomarker for renal functionality (Taylor and Howard, 1989) - demonstrate that the kidney is a target organ for the toxic effects of M8OI in mice after i. p. administration.

### 3.2 Hepatic effects of M8OI

**Fig. 4:**
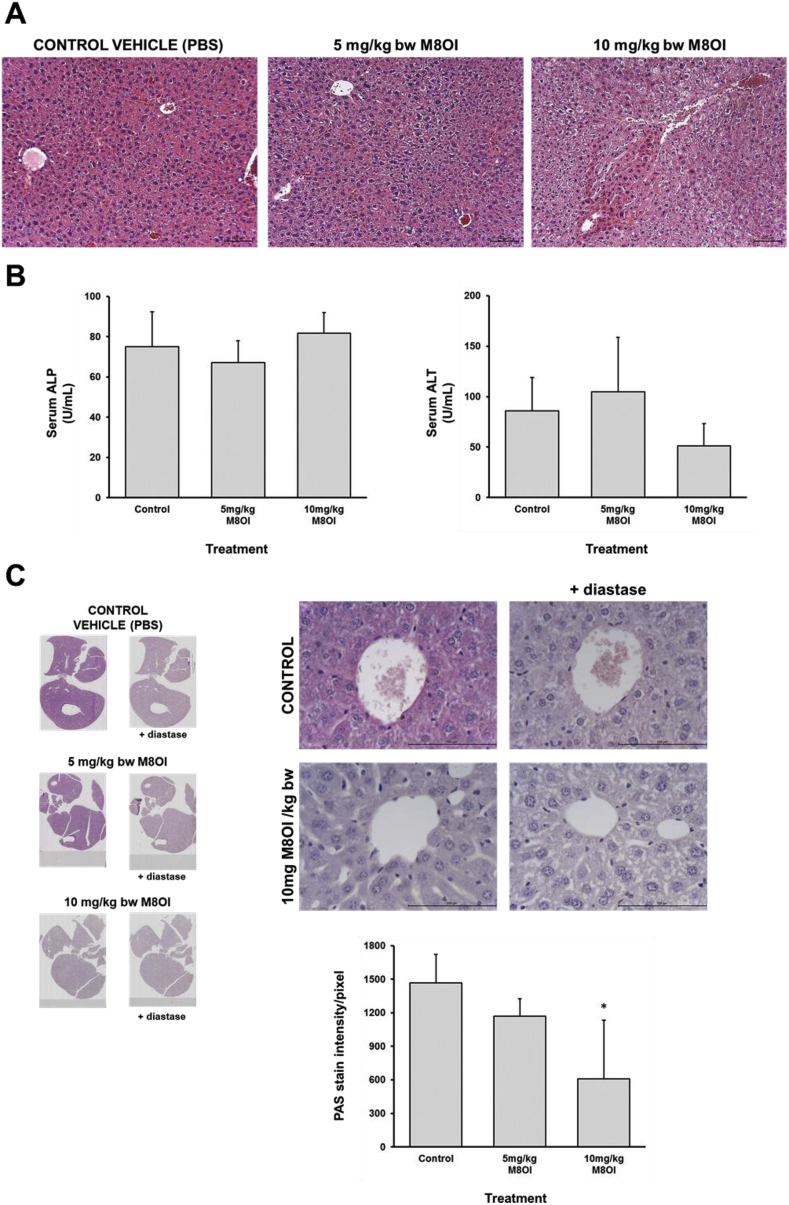
**Lobular effects of M8OI in the liver *in vivo*. A,** typical histopathological views of mouse liver, haematoxylin and eosin stained sections. **B**, left panel, serum ALP; right panel, serum ALT. Data are the mean and SD of at least 3 separate animal serum samples (i.e. not technical replicates) per group. **C**, typical PAS stained liver sections, with as indicated, pre-digestion with diastase: left panel, low magnification displaying entire liver in section; right panel, high magnification. Lower panel, image analysis of PAS stain intensity. Data are the mean and SD of at least 3 separate animal liver section scans (i.e. not technical replicates) per group. *Significantly different (P > 0.95) from control using one way ANOVA followed by bonferroni post hoc test.

Since previous work with the Br-salt of M8OI indicated injury to the liver after exposure via i. p. administration (Yu et al., 2008), livers were examined for evidence of injury and/or change. No overt necrosis of hepatocytes was evident histopathologically although some morphological changes were apparent (Fig. 4a). Negligible hepatocyte necrosis in response to M8OI treatment is supported by an absence in any increases in serum ALT or ALP in response to M8OI exposure at time of termination (Fig. 4b), suggesting an absence for both necrotic hepatocellular and cholestatic liver injury respectively.

**Supplementary Figure 1:**
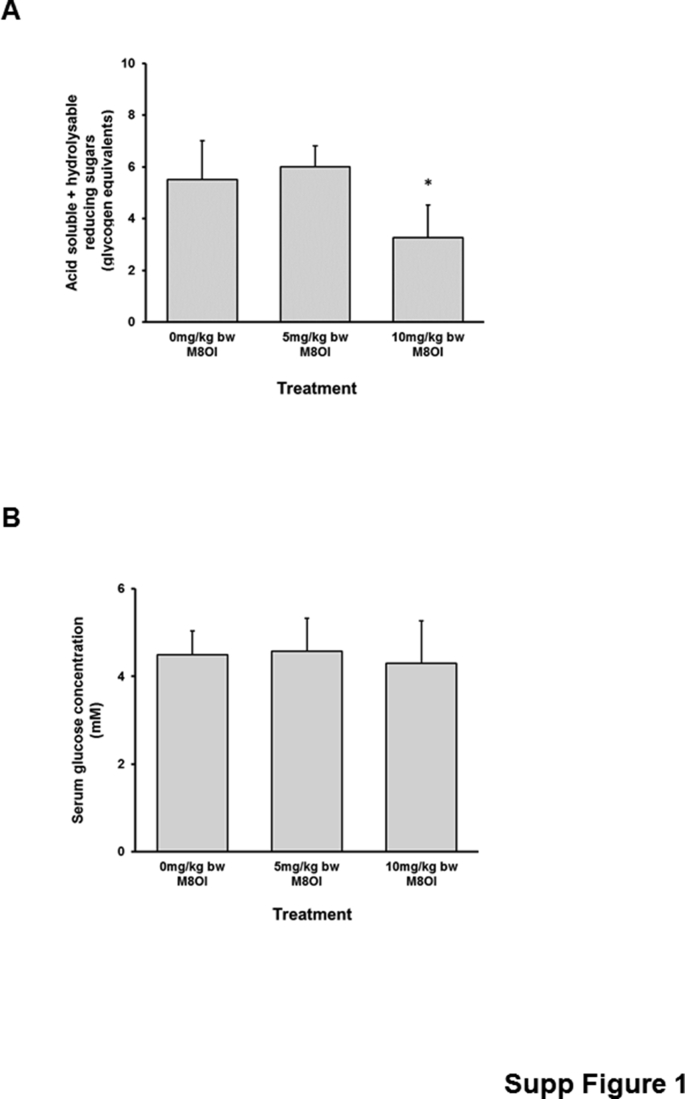
Effects of M8OI on hepatic acid-soluble and hydrolysable reducing sugars and serum glucose levels. A, acid-soluble and hydrolysable reducing sugars where determined and expressed relative to purified glycogen and normalised to total liver protein. B, serum glucose concentrations. All data are the mean and SD of at least 3 separate animals per group. *Significantly different (P > 0.95) from control using one way ANOVA followed by bonferroni post hoc test. .

**Supplementary Figure 2:**
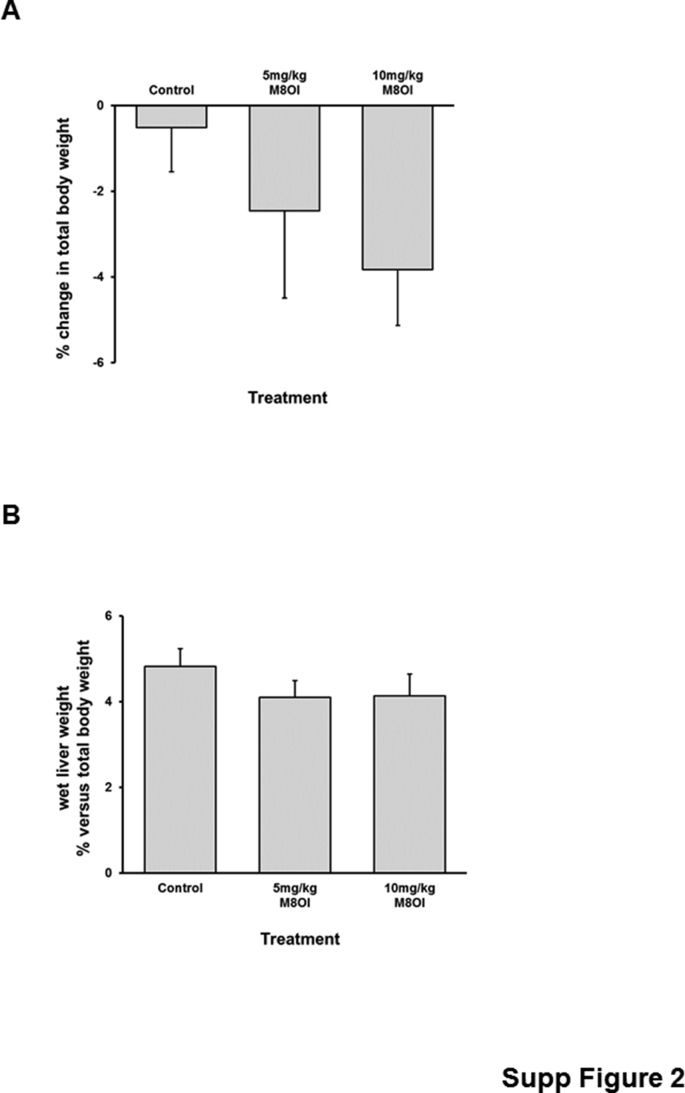
Effects of M8OI on body weight and relative liver weight. A, % change in body weight, expressed relative to starting body weight. B, wet liver weight expressed relative to terminal body weight. All data are the mean and SD of at least 3 separate animals per group. .

Based on the morphological changes observed in sections stained with H & E, it was hypothesised that the changes may in part be associated with a depletion of glycogen. Liver sections were therefore stained by PAS, with and without diastase pre-treatment, and Fig. 4c demonstrates a dose-dependent reduction in glycogen in M8OI-treated mouse liver. Given that the mechanism of toxicity for M8OI is an inhibition of mitochondrial oxidative phosphorylation when cells are exposed to M8OI in vitro (Probert et al., 2018), depletion of liver glycogen suggests that the liver was exposed to M8OI after i. p. administration (Fig. 4c). This was supported by a significant reduction in acid-soluble and acid-hydrolysed reducing sugars (glycogen equivalents) in liver tissue at 10 mg M8OI/kg body weight (Supp Fig. 1a) although this did not manifest in any changes in serum glucose levels (Supp. Fig. 1b). There was a dose-dependent trend for a decrease in body weight and relative liver weight with increasing dose of M8OI but neither reached statistical significance (Supp. Fig. 2).

**Fig. 5:**
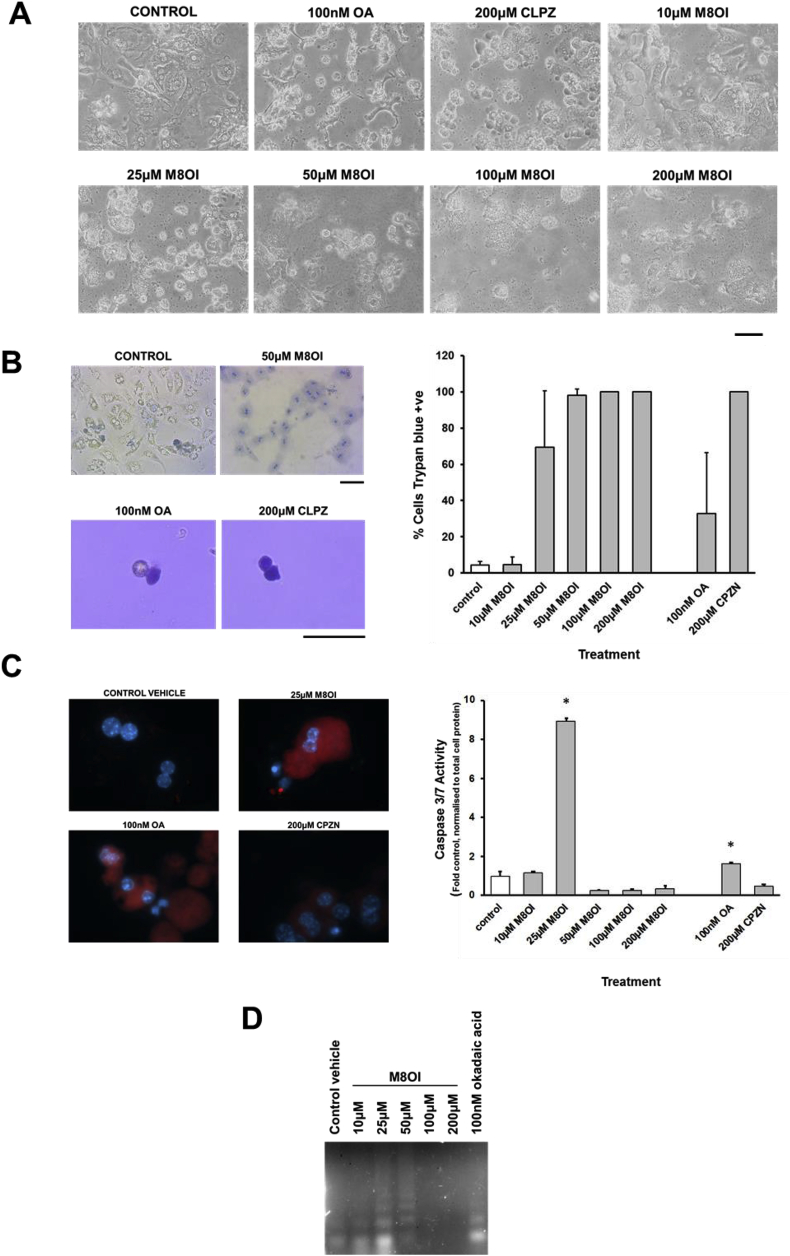

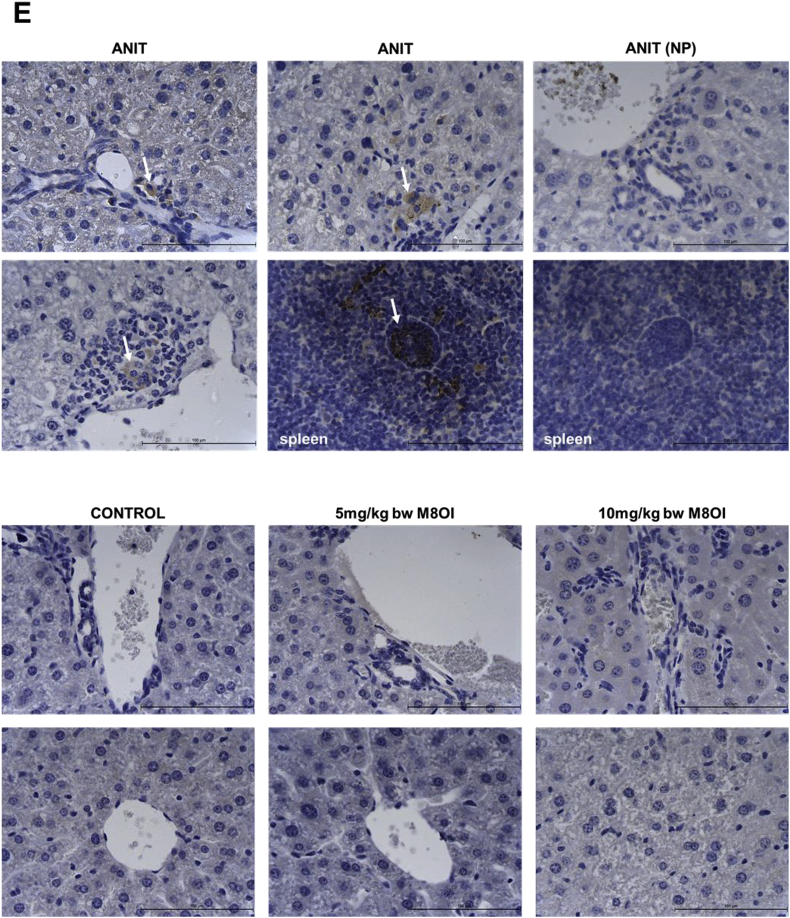
**Mouse hepatocytes undergo an apoptotic mode of cell death in response to M8OI *in vitro*. A**, typical phase contrast images of hepatocytes after treatment with the indicated chemicals for 24 h, except OA and CLPZ where images were taken after 2 h of exposure. Bar = 100 μm. **B**, left panels: photomicrographs of trypan blue-stained hepatocytes treated with the indicated chemicals for 24 h, except OA and CLPZ where images were taken after 2 h of exposure of detached cells. Bar = 100 μm. Right panel: Mean and standard deviation percentage of cells staining positive for trypan blue after 24 h exposure with the indicated chemicals, data are typical of at least 3 separate experiments. For control and M8OI treated cells, data refer to nuclear staining; For OA and CLPZ, data refer to detached cells in suspension after 2 h. **C**, Caspase activity in mouse hepatocytes. Left panel, hepatocytes stained for active caspase 3 (red) and DNA (blue) after treatment with the indicated chemicals for 24 h, except OA and CLPZ where images were taken after 2 h of exposure. Right panel, caspase 3/7 activity – data mean and SD of 3 separate determinations from the same experiment, typical of at least 3 separate experiments. *Significantly different (P > 0.95) from control using one way ANOVA followed by bonferroni post hoc test. **D**, Nucleosomal ladder formation after treatment with the indicated chemicals for 24 h, except OA (2 h). **E**, Immunohistochemical analyses for active caspase 3. Upper panels from mice treated with ANIT, or sections from spleen as indicated, and used as positive controls (arrows indicating cells positive for active caspase 3). ANIT (NP) are sections stained identically except for the addition of anti-active caspase 3 antibody. (For interpretation of the references to colour in this figure legend, the reader is referred to the Web version of this article.)

**Supplementary Figure 3:**
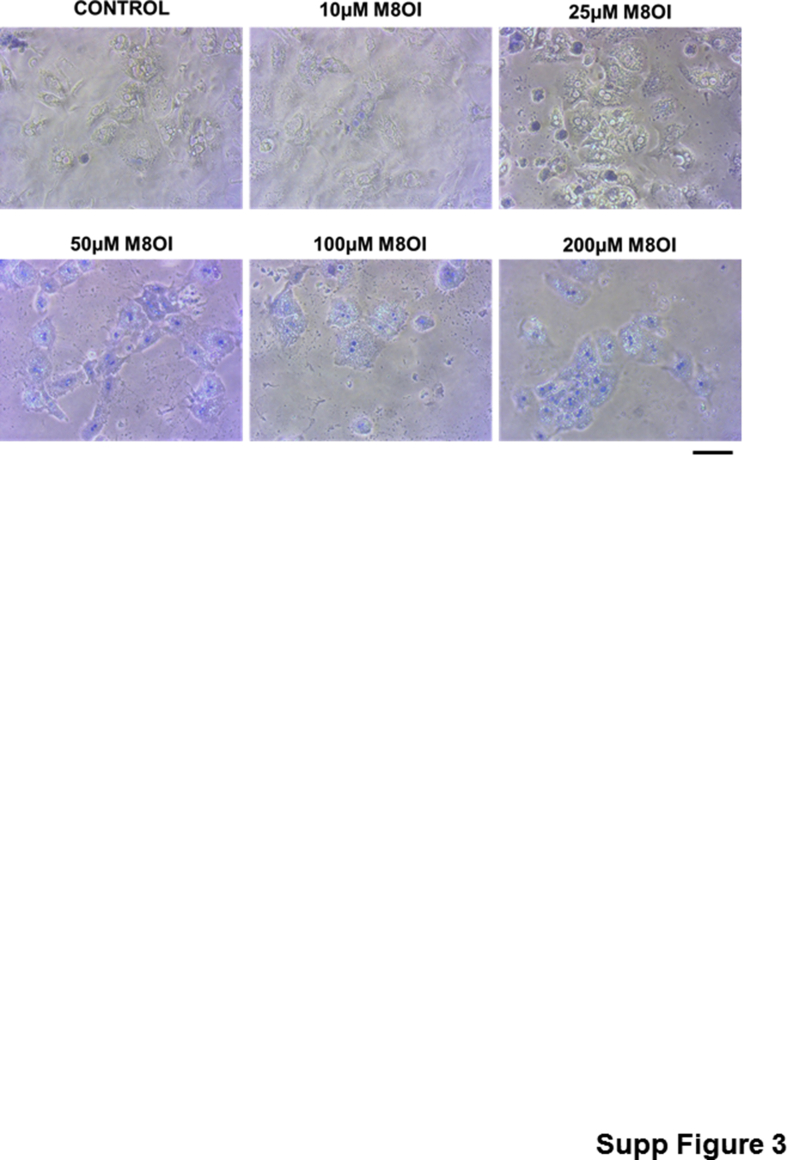
Trypan blue staining of hepatocytes exposed to M8OI. Typical phase contrast images of hepatocytes exposed to trypan blue after treatment with the indicated chemicals for 24 h. Bar = 100 μm. .

Given that M8OI triggered an apoptotic mechanism of cell death in an hepatic progenitor cell line, the toxicity of M8OI in mouse hepatocytes was examined in vitro. Fig. 5a demonstrates that M8OI was toxic to mouse hepatocytes based on morphological changes, with significant changes occurring by 24 h at concentrations between 10 and 25 μM (see also Supp Fig. 3). In contrast, exposing hepatocytes to 100 nM okadaic acid (OA) or 200 μM chlorpromazine (CLPZ) caused marked morphological changes within 1–2 h, with cells rounding and detaching and, for the latter, marked blebbing (Fig. 5a). In contrast, the majority of hepatocytes exposed to toxic concentrations of M8OI did not readily detach. Incubation with trypan blue – which is excluded by intact energised cells (i.e. viable or undergoing programme cell death-related mechanisms of cell death such as early stages of apoptosis) – resulted in a dose-dependent nuclear staining in hepatocytes exposed to toxic concentrations of M8OI (Fig. 5b). OA resulted in a mixed population of detached cells, with the majority excluding trypan blue, whereas CLPZ resulted in complete loss of trypan blue exclusion.

Further, the mechanism of cell death is associated primarily initially with apoptosis based on a cells staining positive for active caspase 3 (Fig. 5c left panels) and a marked and statistically significant increase in active caspase 3/7 activity at 25 μM M8OI (Fig. 5c right panel) and dose-dependent nucleosomal DNA laddering (Fig. 5d). At higher concentrations, the absence of induced active caspase 3/7 activity and a DNA ladder suggest toxicity is manifest as necrosis (Fig. 5c and d).

To determine whether M8OI exposure to mice *in vivo* induce liver cell death through an apoptotic mechanism, liver sections were examined for the presence of active caspase 3. No evidence for an increase in cells containing active caspase 3 above background (Fig. 5e) was found in livers from mice exposed to M8OI - in contrast to ANIT treatment (incorporated as a positive control) - suggesting hepatic concentrations of M8OI were not sufficient to induce apoptosis *in vivo*.

**Fig. 6:**
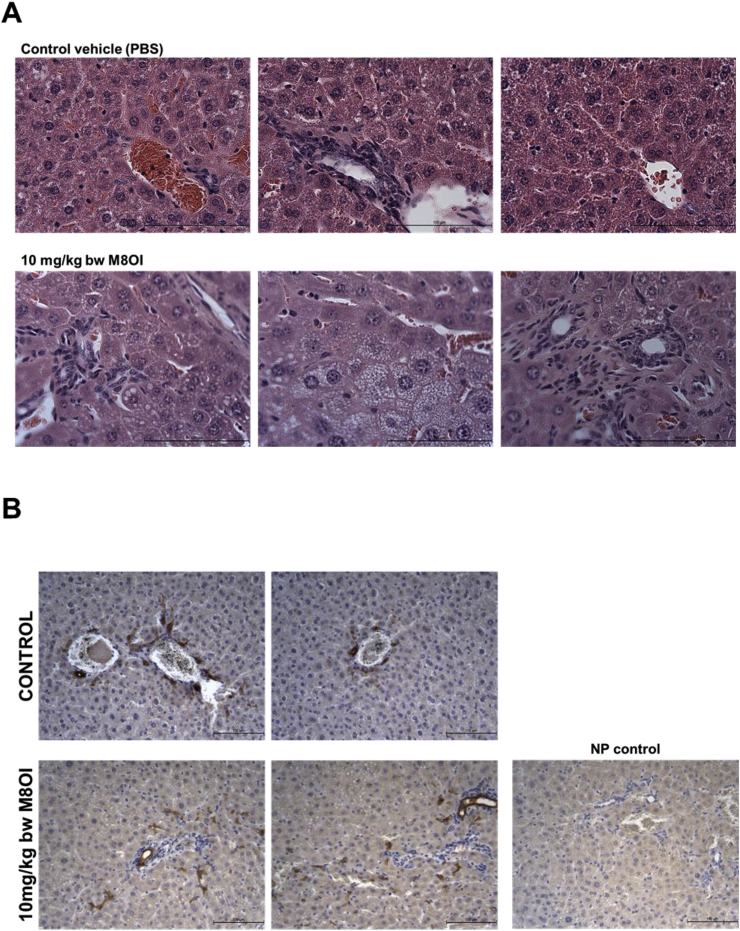

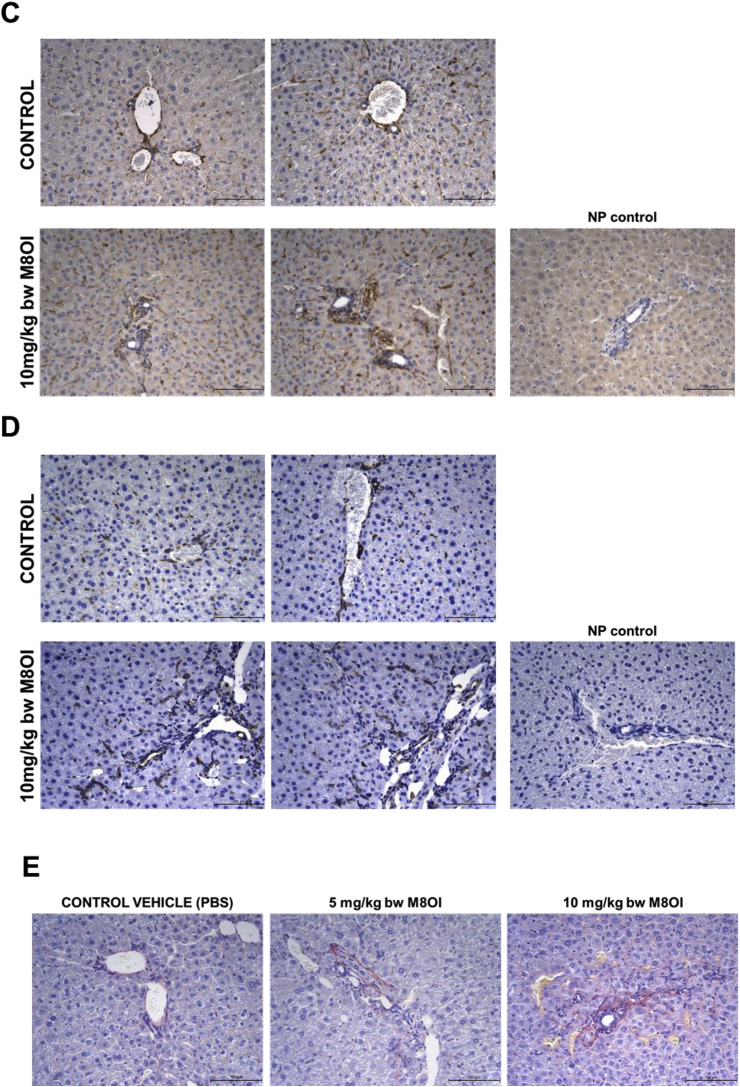

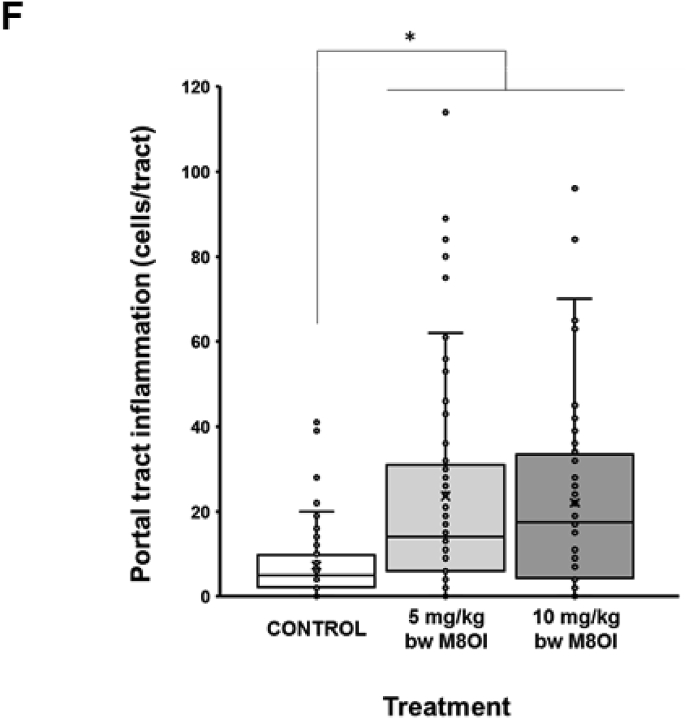
**Portal tract effects of M8OI in the liver *in vivo*. A,** typical histopathological high-powered views of mouse liver, haematoxylin and eosin stained sections. **B**, typical views for CK-19 immunohistochemistry in liver sections from the indicated groups. **C**, typical views for vimentin immunohistochemistry in liver sections from the indicated groups. **D**, typical views for α-sma immunohistochemistry in liver sections from the indicated groups. **E**, typical sirius red stained liver sections from the indicated groups. **F**, dot plot of duct-associated inflammatory cells were counted around least 18 and up to 24 randomly-selected duct regions by an examiner blinded to the identity of the section. Data from 3 animals per group. *Significantly different (P > 0.95) from control using one way ANOVA followed by bonferroni post hoc test. (For interpretation of the references to colour in this figure legend, the reader is referred to the Web version of this article.)

Examination of liver sections stained with H&E for changes other than overt cell death indicates some inflammatory cell recruitment and a mild degree of ductular hyperplasia (Fig. 6a). These regions stained positive for the cholangiocyte marker CK-19 (Fig. 6b) and were associated with both fibrogenic vimentin-positive fibroblasts (Fig. 6c), α-smooth muscle actin positive myofibroblasts (Fig. 6d) and a mild accumulation of sirius red-positive collagenous matrix accumulation (Fig. 6e). Given that normal rodent liver often shows variable background levels of patchy ductular inflammatory cell recruitment, haematoxylin and eosin-stained sections were scored for duct-associated inflammatory and fibroblastic cells with at least 18 and up to 24 duct regions counted per section. Fig. 6f demonstrates the high degree of intra-group variability between mice, however, also that there was a dose-dependent increase in ductular inflammatory and fibroblastic cell recruitment in response to M8OI.

### 3.3 Mouse kidney tissue slices accumulate higher levels of M8OI and injury than liver tissue slices in culture

**Fig. 7:**
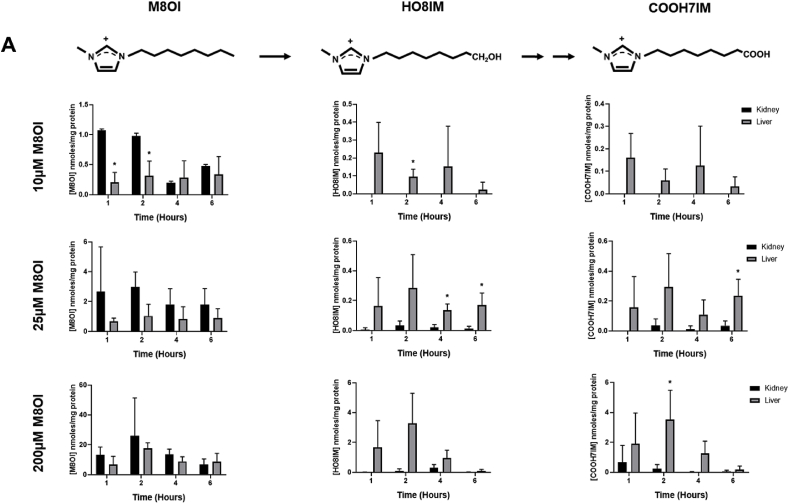

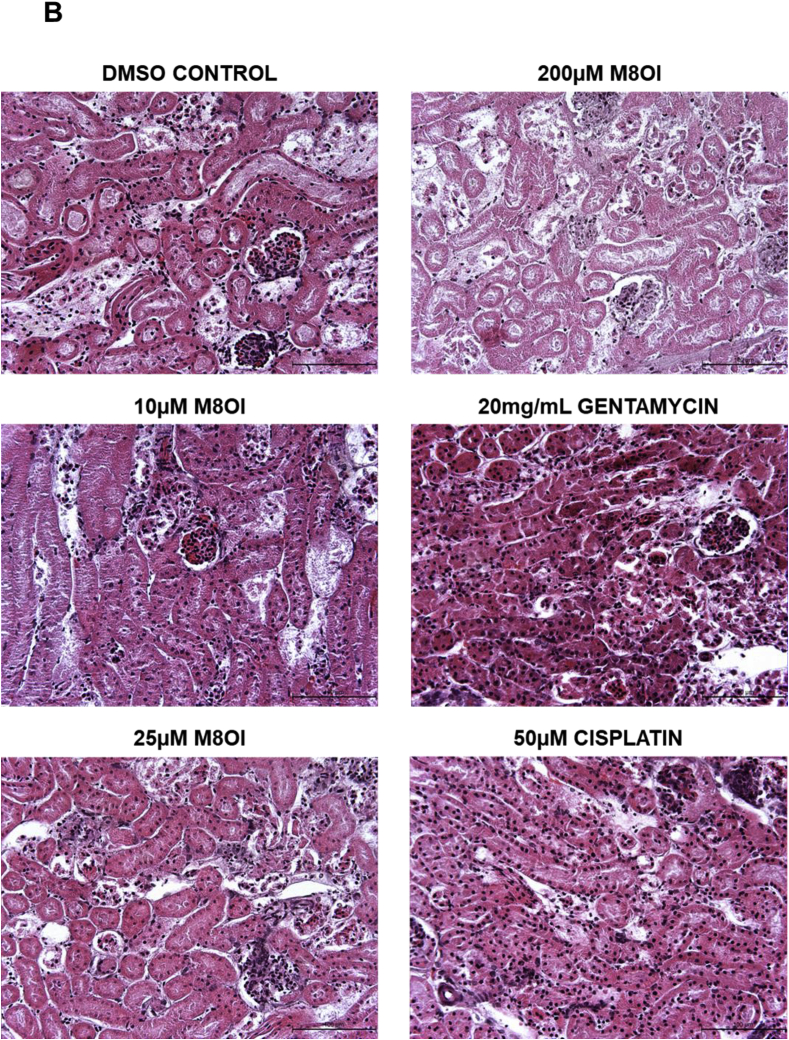

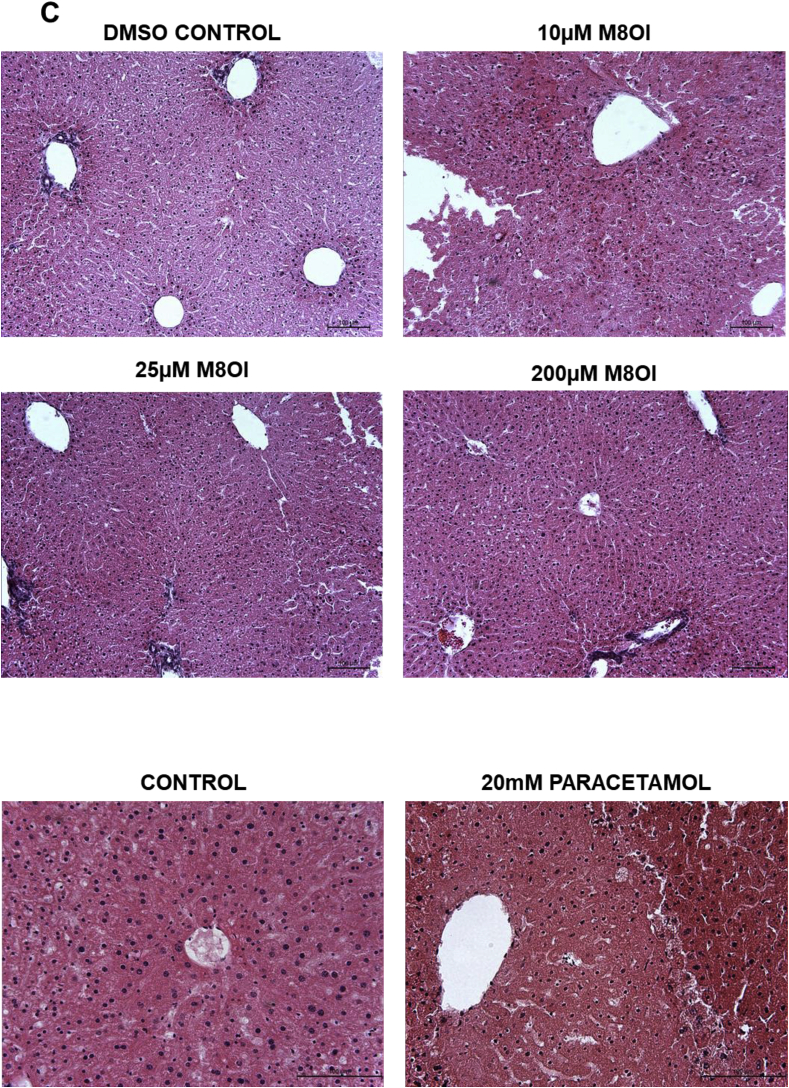
**Disposition and toxicity of M8OI in cultured mouse kidney and liver slices. A,** tissue levels of M8OI and the hydoxylated metabolite HO8IM and carboylated metabolite COOH7IM. Kidney or liver slices were incubated with the indicated concentration of M8OI prior to tissue level determinations. Tissue concentrations are expressed relative to total slice protein. Data are the mean and standard deviation of 3 separate experiments. *Significantly different (p < 0.05) versus equivalent treatments in kidney slices using Student's t-test (two-tailed)**. B,** typical histopathological high-powered views of mouse kidney tissue slices treated for 24 h with the indicated concentration of M8OI or drug, haematoxylin and eosin stained. **C,** typical histopathological high-powered views of mouse liver tissue slices treated for 24 h with the indicated concentration of M8OI or drug, haematoxylin and eosin stained.

In order to determine the potential mechanism(s) underpinning target kidney injury in response to M8OI in the mouse, liver and kidney tissue slices were exposed to increasing concentrations of M8OI in culture and the levels of tissue M8OI determined for up to 6 h. Fig. 7a demonstrates that higher levels of M8OI accumulate (up to greater than 4 fold higher when incubated with 10 μM M8OI) within kidney slice tissue after 1 h at all doses, followed by a fall, with low levels of metabolites detected. In contrast, liver tissue slice levels of M8OI were lower than kidney levels and remained relatively stable, with additionally, significant metabolism of M8OI to its hydroxylated and carboxylated metabolites HO8IM and COOH7IM respectively (for structures, see Fig. 7a).

Examination of tissue sections after 24 h of exposure to M8OI demonstrates that the mouse kidney slices showed dose-dependent necrotic changes (Fig. 7b), as observed with established nephrotoxins gentamycin (Girton et al., 2002) or cisplatin (Wainford et al., 2008). Despite centrilobular injury in mouse liver slices in response to high concentrations of paracetamol (Jaeschke et al., 2018), treatment with M8OI did not cause any apparent necrotic (or apoptotic) effects (Fig. 7c).

## 4 Discussion

This paper is the first - to our knowledge – to examine the potential toxicity of M8OI in several organs in a mammalian species *in vivo*. To avoid any potential interactions of counter-ion toxicity, the effects of chloride salt (M8OI.Cl, i.e. the common physiological chloride anion) was used. The single paper in which M8OI toxicity has been examined in a mammalian species *in vivo*, is with the bromide salt (Yu et al., 2008). This study was limited in scope, focussing solely on any acute liver effects in the mouse after a single i. p. injection up to 40 mg/kg body weight. The authors report an LD_50%_ of 35.7 mg/kg for M8OI [Br^−^], a marked histopathological change in the liver at 10 h (apparent cytoplasmic vacuoles) and small changes in the activity of some ROS-related endpoints though it is not clear what role these changes have in the mechanism of liver injury.

Bromide represents approximately 29.1% by weight of M8OI [Br^−^] and therefore, at the maximum dose used, exposure to bromide would be 11.6 mg/kg body weight. Bromide salts have been used clinically in the past with toxic blood levels (causing mental and neurological disturbances) reported at blood levels of 1–3 g/L (, European Commission, 2000). Mice fed diets containing sodium bromide at a level of 0, 400, 1200, 3600 or 10,800 ppm for 36 days (equivalent to 0, 80, 240, 720 or 2160 mg/kg body weight) resulted in behavioural and body weight changes between 400 and 1200 ppm (Hansen and Hübner, 1983). Taking a conservative approach regarding dose (NOAEL of 80 mg/kg body weight and that these effects required 36 days of continuous exposure), and given that an i. p. exposure is a bolus administration, it is unlikely that the single i. p. bolus administration at the maximum dose of 40 mg M8OI [Br^−^]/kg body weight was toxic due to bromide or likely significantly influenced by the bromide cation. Therefore, the effects of M8OI [Br^−^] in the study of Yu et al. (2008) are likely associated with the M8OI moiety rather than bromide. However, given that these effects were reported in the LD_50%_ range, the hepatic effects may have been influenced by the high (lethal) doses used and significantly associated with extra-hepatic non-specific effects associated with multi-organ failure.

This study indicates that the primary target organ for the toxic effects of M8OI is the kidney, based on both histopathological alterations and changes in serum and urinary parameters associated with kidney injury. Given that the proposed mechanism by which M8OI interacts with cells is via inhibition of oxidative phosphorylation in mitochondria, any effects may be associated with metabolic links between multiple organs. Alternatively, the kidney may be selectively sensitive to M8OI due to its reliance on cellular respiration for reabsorption. For example, although constituting only 0.5% of body mass, kidneys consume 10% of the oxygen used in cellular respiration (Berg et al., 2002). An additional consideration is that the kidney may be a significant route for elimination of M8OI and therefore, in the process of elimination, exposed to relatively high intracellular concentrations of M8OI. Given the apparent resistance of the heart to injury from M8OI (which is similarly highly dependent on mitochondrial function for high ATP generation/turnover), this aspect may be particularly significant in the mouse.

The addition of M8OI to cultured kidney and liver tissue slices resulted in uptake of M8OI in both tissues, with levels of accumulation higher in kidney at early time points in particular. The higher peak concentration of M8OI and limited metabolism by kidney slices may therefore be significant for M8OI toxicity, which was observed in treated kidney, but not liver slices. Interestingly, there was accumulation of M8OI followed by an apparent loss or excretion by 6 h in kidney slices. This may suggest that the disposition of M8OI may be heavily influenced by active transport in the kidney. However, the interactions of M8OI or its metabolites with transporters – if any - is currently unknown. Furthermore, it cannot be excluded that this apparent fall in kidney M8OI levels is not associated with some unknown metabolic transformation.

There is no evidence in the current study for hepatocyte necrosis from histopathological examination or from clinical chemistry. However, mouse hepatocytes in vitro appear to be relatively sensitive to M8OI and to undergo apoptosis, as observed for other cell types (Probert et al., 2018). Given the absence of any evidence for apoptosis in the liver *in vivo* at the highest dose used in this study, it is likely that threshold liver concentrations of M8OI capable of inducing apoptosis were not reached despite evidence for hepatic exposure (i.e. glycogen depletion). It is therefore possible that the hepatic effects seen by Yu et al. (2008) with 37.5 mg/kg body weight were associated, to some extent, with widespread hepatocyte apoptosis although this endpoint was not examined. The portal tract changes identified as a mild hepatic effect in this study is an additional effect that also appears to have occurred in the absence of any hepatic necrotic or apoptotic injury. The cause of this effect is unknown but may be associated with its ability to activate the mouse estrogen (ER) receptors, including super-activation of the mouse ER-β variant 2 receptor (Leitch et al., 2018). Estrogens are known to be cholestatic (Stieger et al., 2000; Yamamoto et al., 2006), however, cholestatic injuries are normally accompanied by elevations in serum markers such as ALP (Meyer et al., 2017) and this was not observed in this study.

Ionic liquids show great potential in a variety of industrial applications and have the advantage of offering an alternative – more environmentally friendly – option to the use of volatile solvents. Despite this, there are limited data regarding their potential toxicity, in particular in mammalian systems. This report demonstrates for the first time, that the kidney is a target organ for the acute toxic effects of M8OI and that the liver may be a target for some changes. The route of exposure (i.p.) was adopted in order to maximise detection of target organ effects whilst reducing the number of animals used and avoiding animal suffering and was not designed as a study to assess risk to human health. The most realistic route of exposure in man will be the oral route (and dermal route for occupational exposure). A chronic study employing an oral route of exposure to M8OI may help to clarify any potential renal, hepatic and other organ hazards in man.

## Credit author statement

Alistair Leitch, Tarek Abdelghany: Conceptualization, Methodology, review, analysis, Data curation and writing. Alex Charlton, Justina Grigalyte, Fiona Oakley, Lee Borthwick, Lee Reed, Amber Knox, William Reilly, Loranne Agius: Methodology, review, analysis. Peter Blain, Matthew Wright: Conceptualization, Methodology, review, analysis, funding, Supervision and editing, writing.

## Declaration of competing interest

The authors declare that they have no known competing financial interests or personal relationships that could have appeared to influence the work reported in this paper.
